# Evaluation of a pilot implementation of a digital cognitive behavioral therapy platform for isolated older adults in county mental health services

**DOI:** 10.1177/26334895241288571

**Published:** 2024-10-15

**Authors:** Rosa Hernandez-Ramos, Stephen M. Schueller, Judith Borghouts, Kristina Palomares, Elizabeth Eikey, Margaret Schneider, Nicole A. Stadnick, Kai Zheng, Dana B. Mukamel, Dara H. Sorkin

**Affiliations:** 1 Department of Psychological Science, University of California, Irvine, CA, USA; 2 Department of Informatics, University of California, Irvine, CA, USA; 3 Department of Medicine, 8788 University of California Irvine, Irvine, CA, USA; 4 Herbert Wertheim School of Public Health and Human Longevity Science, 8784 University of California, San Diego, La Jolla, CA, USA; 5 Department of Public Health, University of California, Irvine, CA, USA; 6 Department of Psychiatry, 8784 University of California, San Diego, La Jolla, CA, USA; 7 Altman Clinical and Translational Research Institute, Dissemination and Implementation Science Center, 8784 University of California, San Diego, La Jolla, CA, USA; 8 229241 Child and Adolescent Services Research Center, San Diego, CA, USA

**Keywords:** county mental health, isolated older adults, pilot implementation, RE-AIM, technology-enabled services

## Abstract

**Background:**

Technology-enabled services (TESs) have the potential to increase access to mental healthcare. However, little research has focused on how TESs can be integrated into publicly funded service settings. As part of the state-wide Help@Hand project, Marin County conducted a pilot implementation of myStrength, a digital cognitive behavioral therapy platform, to explore its potential to reduce loneliness among isolated older adults. We evaluated the pilot impact using the Reach, Effectiveness, Adoption, Implementation, and Maintenance (RE-AIM) framework.

**Method:**

A single-site 6-month pilot implementation recruited English (*n* = 15) and Spanish-speaking (*n* = 15) isolated older adults who received a digital literacy course followed by 8 weeks of myStrength access and human support. We evaluated factors related to reach, effectiveness, adoption, and implementation using the perspectives of users and County staff. Descriptive statistics were used to examine reach, adoption, and implementation. Nonparametric tests, including Friedman and Wilcoxon signed-rank, were used to examine effectiveness.

**Results:**

Reach: Compared to overall county demographics, platform users were majority female (93.1% vs. 50.5%), ethnoracialized (62.1% vs. 24.2%), and of lower socioeconomic status (*Mdn* = $35,000 vs. $131,008). Effectiveness: Users reported a significant (*z* = −2.62, *p* < .001) decrease in loneliness. Adoption: Users logged into myStrength an average of 10 times and completed 33 activities during the 8 weeks of myStrength use. Implementation: Each pilot staff (*N* = 20) spent an average of 19.8 hr (*SD* = 16.51) supporting users’ use of myStrength during the pilot. Pilot staff reported several adaptations to meet the needs of users.

**Conclusions:**

Successes included reaching the target population, reducing loneliness, and user adoption. However, pilot staff invested significant time to support those with lower digital literacy skills. As such, although TESs may address unmet needs, their use with underserved populations may require upfront and ongoing support provided by the settings where they are implemented.

**Plain Language Summary Title:**

Testing a New Digital Therapy Tool for Isolated Older Adults in County Mental Health Services.

## Plain Language Summary

This paper reports on the use of digital tools to support needs in publicly funded mental healthcare. In 2021, Marin County piloted a program involving the use of myStrength, a digital cognitive-behavioral therapy (dCBT) platform, with human support provided by a nurse intern or promotores. Although several studies have demonstrated that dCBT can be effective, few studies have evaluated this in real-world settings or considered issues related to its implementation. As such, although dCBT can provide benefit to people, less is known about how to deploy it, especially in diverse contexts and populations. This project specifically focused on reducing loneliness among isolated older adults, including both English and Spanish speakers. This paper adds valuable insights by demonstrating that the project successfully reached the target population, reduced loneliness, and achieved high user adoption rates. It also identified challenges, including low digital literacy skills among the target populations and the need for substantial upfront and ongoing support. The findings suggest that the combination of dCBT and human support holds promise in addressing unmet mental health needs, especially among vulnerable populations. At the same time, the implementation of these services in such contexts demands substantial resources and support, especially to bridge the digital literacy gap. These implications have practical significance for practitioners, researchers, and policymakers, emphasizing the importance of tailored support mechanisms when deploying digital tools in publicly funded service settings to ensure equitable access and effectiveness.

## Introduction

Loneliness constitutes a public health crisis for the U.S. aging population (U.S. Department of Health and Human Services, 2023), with approximately 43% older adults reporting feeling lonely (National Academies of Sciences, Engineering and Medicine, 2020). This is alarming given that decades of research have shown that loneliness is associated with adverse health outcomes (Holt-Lunstad, 2021).

Previous research has underscored the effectiveness of cognitive behavioral therapy (CBT) in treating loneliness (Hickin et al., 2021). However, among older adults with mental health concerns, more than half do not receive services due to logistical barriers, including cost (SAMHSA, 2020). Technology-enabled services (TESs)—digital translations of psychological interventions paired with human support—may broaden access to evidence-based interventions, including CBT, given that they minimize the need for specialty mental health providers.

Although current evidence has concluded that both digital CBT (dCBT) and TESs are efficacious (Karyotaki et al., 2021; Werntz et al., 2023), more work is needed on how they can be deployed, including within publicly funded service settings. Accordingly, this paper reports on the opportunities and challenges from a county-led pilot implementation of myStrength, a dCBT platform, for isolated older adults. Results are organized by the Reach, Effectiveness, Adoption, Implementation, and Maintenance (RE-AIM) framework (Glasgow et al., 2019).

## Method

### Design

**Figure 1:**
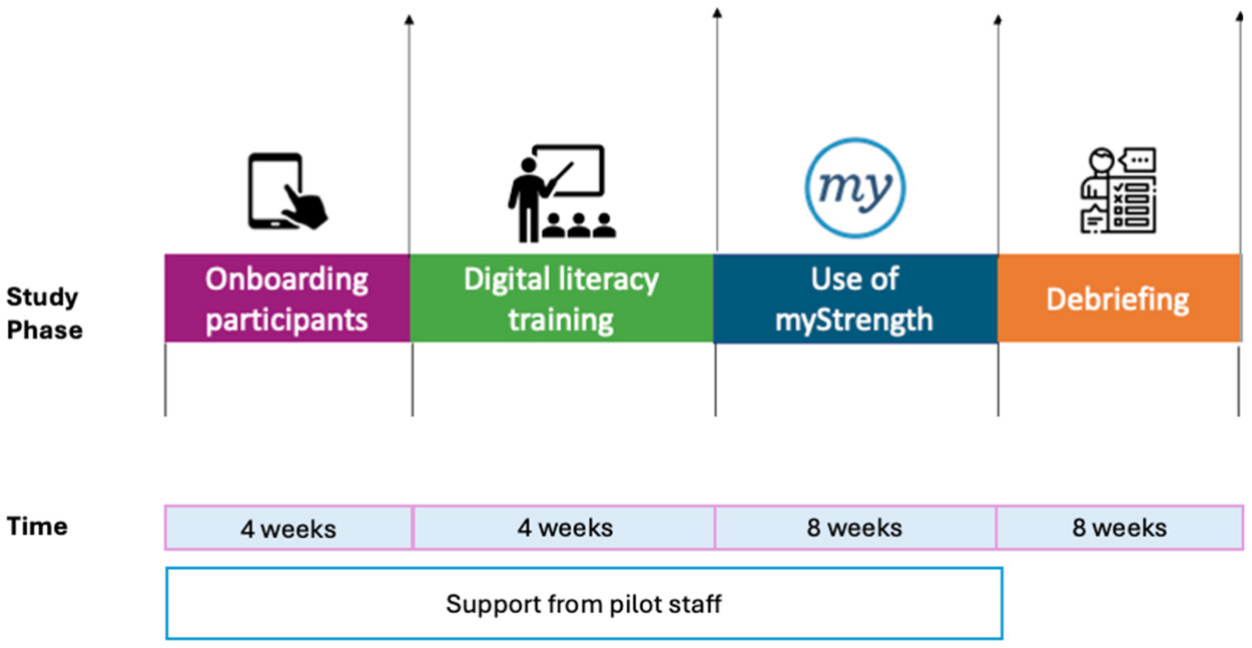
Overview of Pilot Timeline.

As part of the California Mental Health Services Authority's “Help@Hand” multi-county technology innovation project (CALMHSA, 2024), Marin County conducted a single-site pilot implementation of myStrength with an embedded process evaluation conducted by our research team.^1^ Figure 1 shows a detailed timeline of the pilot. The University of California, Irvine, Institutional Review Board approved the evaluation activities (review number 20195406).

### Participants

#### Platform Users

Marin County sought to recruit underserved isolated^2^ (e.g., geographically, culturally, and socially) older adults. To recruit this target population, Marin County enlisted the help of existing community partnerships working with isolated older adults, including the Telehealth Equity Project, the West Marin Senior Services, Meals on Wheels, and an established network of promotores (i.e., community health workers). Individuals were eligible for participation if they were aged 60 years or older and were able to speak, read, and write in English or Spanish. Those with physical or mental challenges that would impair participation were deemed ineligible. Prior experience with technology was not a requirement for participation. Eligible individuals^3^ (*N *= 30; English speakers: *n *= 15, Spanish speakers: *n *= 15) who provided written informed consent were enrolled as platform users.

#### Pilot Staff

Several pilot-specific staff (*N *= 20), including nurse interns (*n *= 13), promotores (*n *= 4), and county project leadership (*n *= 3), self-selected to this pilot implementation. Nurse interns were recruited from two local universities. Promotores were recruited from an established network of county volunteers. Staff did not have previous experience with myStrength.

### Procedure

#### Digital Literacy Training Course

Participants were invited to an optional group-based four-class (each 2 hr) digital literacy training held via Zoom. The training was developed in collaboration with a local community organization whose mission is to teach adults of all ages how to use technology. Classes covered computer basics, Internet basics, email basics, and an introduction to myStrength.

#### myStrength Platform

Users were provided with no-cost access for 8 weeks to myStrength,^4^ a dCBT platform for the self-management of symptoms of depression, anxiety, stress, substance use disorder, chronic pain, and sleep problems (myStrength, 2022). They were encouraged to use the platform at their discretion, without specific guidelines on frequency. The platform, which includes psychoeducation, mood tracking, community forums, and interactive activities, has been shown to be effective in increasing life functioning, and reducing depression and stress (Hirsch et al., 2011, 2017; Schladweiler et al., 2017).^5^

#### Human Support

All users were offered human support starting at onboarding with assistance in setting up Wi-Fi and devices. During the use period, staff conducted weekly phone calls with users. These calls allowed staff to address any technological issues, answer any content questions, and encourage use of myStrength. Pilot staff were not provided with specific guidelines regarding the duration of these phone calls; instead, they were encouraged to use their discretion.

### Measures

Users completed surveys at three time points. Users who attended at least one class of the digital literacy training course (*n *= 28) were asked to complete measures before and after the course (Surveys 1 and 2). All users (*N *= 30) were asked to complete measures before and after their 8-week use period (Surveys 2 and 3). Users received a $10 gift card for each completed survey. Staff completed one survey at the end of the pilot. Due to organizational policy, only promotores were able to receive a $15 gift card for completing the survey. Socio-demographic variables were collected at the first completed survey time point for each respondent. Surveys were completed online or via a phone call, depending on respondent preference.

#### Reach

To determine reach, demographics of recruited users were compared to the demographics of older adults (>65 years) in Marin County, using aggregated data from the U.S. Census Bureau and the California Department of Aging (California Department of Aging, 2020; U.S. Census Bureau, 2022).

#### Effectiveness

To determine the effectiveness, change in users’ loneliness was assessed using the UCLA Three-item Loneliness Scale (Hughes et al., 2004). The scale includes three statements that each measure one of the three dimensions of loneliness, including relational connectedness, social connectedness, and self-perceived isolation, rated on a 3-point Likert scale—1 (*hardly ever*), 2 (*some of the time*), and 3 (*often*). Total scores range from 3 to 9, with higher scores indicating greater levels of loneliness. The scale has been shown to have high reliability, convergent validity, and construct validity in both English-speaking and Spanish-speaking samples (Russell et al., 1980; Trucharte et al., 2023). Our Cronbach’s alpha ranged from α = .66 to .85.

#### Individual-Level Adoption

To assess individual-level adoption, we evaluated user engagement by measuring the average number of logins and completed activities, using platform data provided by the myStrength vendor.

#### Implementation

To understand organizational aspects that emerged during the pilot implementation of myStrength, we assessed adaptations and related costs.

Adaptations were assessed using a three-item scale developed by our team, based on the Framework for Reporting Adaptations and Modifications-Expanded for tracking intervention modifications (Stirman et al., 2019). Staff were asked to rate the extent to which they agreed or disagreed with three statements on a 5-point Likert scale, ranging from 1 (*completely disagree*) to 5 (*completely agree*). For instance, “I integrated supplemental content or strategies when I discussed myStrength with my clients.” We summed the scores for the three items producing a total score ranging from 3 to 15, with higher scores indicating higher amounts of adaptations. Our Cronbach’s alpha was α = .68.

Related costs were measured by assessing the time staff, which was self-reported and tracked throughout the course of the 6-month pilot, spent providing direct human support. We measured related costs in terms of time and not monetary value because our team did not have access to data relating to staff salaries.

### Data Analysis

Given the recruited sample size, data were analyzed using descriptive and nonparametric statistics in SPSS Version 29. Missing data due to either attrition or nonresponse were handled using case-wise deletion for each collected measure. Descriptive statistics including measures of central tendency, frequency counts, and percentages were calculated and reported for reach, adoption, and implementation. To evaluate the effectiveness of myStrength, we used the omnibus Friedman test to assess within-subject changes in loneliness scores of users, overtime. In case of significant omnibus finding (α = .05), we planned on following established guidelines and carry out post hoc analyses with Wilcoxon signed rank tests for paired samples to test for pairwise comparisons (Pereira et al., 2015; Scheff, 2016). To adjust our family-wise alpha level so it remained at our target of α = .05, we used a Dunn-Bonferroni correction to partition the αFW across the three contrasts (αPC = .016).

## Results

### Reach

The sociodemographic characteristics of users are shown in Table 1. As shown in Table 2, when descriptively comparing the available demographics of our sample of users to Marin County demographics, our sample was composed of more females, fewer English speakers, individuals with higher levels of education, a more diverse ethnoracial composition, and individuals with lower incomes.

**Table 1:** Sociodemographic Characteristics of Platform Users.

Characteristics	Full sample		Effectiveness sample	
	*n*	%	*n*	%
Sex				
Female	27	93.1	18	90.0
Preferred language				
English	14	48.3	6	30.0
Spanish	15	51.7	14	70.0
Ethnicity				
Black	1	3.5	1	5
Latine	16	55.1	15	75.0
White	11	37.9	5	25.0
Biracial	1	3.5	0	0
Highest educational level^a^				
High school or less	8	27.6	7	35.0
More than high school	18	62.1	13	65.0
Household income^b^				
Less than $19,000	12	41.4	9	45.0
$20,000–$39,000	3	10.3	3	15.0
$40,000–$59,000	2	6.9	1	5.0
$60,000–$79,999	2	6.9	0	0
More than $80,000	4	13.8	7	35.0
Employment status^c^				
Full-time	1	3.5	1	5.0
Part-time	1	3.5	2	10.0
Unemployed	5	17.2	5	25.0
Retired	11	37.9	6	30.0
Disabled	6	20.7	6	30.0
Marital status^d^				
Unpartnered	19	65.5	12	60.0
Partnered	9	31.0	8	40.0
Living situation^e^				
Lives alone	8	27.6	4	20.0
Lives with others	17	58.6	16	80.0
Mental health concerns^f^				
Experienced	11	37.9	5	25.0
Did not experience	12	41.4	15	75.0
Disability	14	48.3	10	50.0
Has health insurance	25	86.2	18	90.0
Has insurance that cover mental health	15	51.7	13	65.0
Technical readiness prior to pilot initiation				
Needed support getting access to Wi-Fi	6	20.7	4	20.0
Never access the Internet	6	20.7	4	20.0
Not confident using technology	21	72.4	17	85.0

*Note. N* = 29 for full sample as sociodemographic data is missing for one participant user, who chose not to complete a demographic survey. The average age for the full sample was 72 (*SD *= 7.8; range, 60–89). The sample size for effectiveness outcomes was *n* = 20 after accounting for attrition and nonresponse. The average age for the effectiveness sample was 70 (*SD *= 7.5; range, 60–89).

^a^ Three platform users preferred not to answer this question.

^b^ Six platform users preferred not to answer this question.

^c^ Five platform users selected “other” for this question but did not provide specifics.

^d^ One participant user preferred not to answer this question.

^e^ Four platform users selected “other” for this question but did not provide specifics.

^f^ Two platform users selected “other” for this question but did not provide specifics; four platform users preferred not to answer this question.

**Table 2:** Descriptive Comparison of Pilot Sample and Population Sociodemographic Characteristics.

Characteristics	Our sample	Marin county
	%	
Age (over 65 years)	93.1	16.8
Sex		
Female	93.1	50.5
Preferred language		
English	48.3	86.4
Other than English	51.7	13.6
Race		
White	37.9	75.8
Black	3.5	13.6
Hispanic or Latine	55.1	18.9
Highest educational level		
High school or less	27.6	66.3
College degree or more (AA, BA, MA, PhD)	62.1	33.7
Living situation, %		
Lives alone	27.6	23.7
Lives with others	58.6	76.3
Household income (*Mdn*)	$35,000	$131,008

### Effectiveness

Data from 20 users were available at all three time points to determine effectiveness.^6^ Loneliness was significantly different across the three time points, χ^2^(2, *n *= 20) = 6.9, *p *= .03. The effect size was small with a Kendall's *W* of .17. Follow-up pairwise comparisons significant difference in loneliness between Surveys 1 (*Mdn *= 6.00) and 3 (*Mdn *= 5.00), *z *= −2.62, *p *< .001 but not between Surveys 1 and 2, *z *= −1.43, *p *= .15 or Surveys 2 and 3, *z *= −1.48, *p *= .14. The effect size was medium with a Pearson's *r *= .58, 95% CI [0.18, 0.81].

### Adoption

All 30 users logged into the platform at least once, over the course of the 8-week use period. On average, users logged into myStrength 10 times (*SD *= 11.7, range = 1–59). Seventeen out of 30 users completed at least one activity averaging 33 completions (*SD *= 53.5, range* *= 1–198) over the course of the 8-week use period.

### Implementation

#### Adaptations

Data from 19 pilot staff (Table 3 contains socio-demographics) revealed an average score of 10.67 (*SD *= 2.02; range = 7–14), on our three-item scale, which was above the midpoint (*Mdn *= 6.00) of the overall scale indicating staff made several modifications to their interactions with users regarding myStrength. A breakdown of answers by question is in Supplementary Appendix A.

**Table 3:** Sociodemographic Characteristics of Participant Staff at Debriefing.

Characteristics	Full sample	
	*n*	%
Role		
Nurse interns	13	65.0
Promotores	4	20.0
In-house county staff	3	15.0
Sex		
Female	16	84.2
Ethnicity		
White	1	5.3
American Indian/Alaskan Indian	8	42.2
Multiracial	3	15.8
Hispanic or Latino	7	36.8
Highest educational level		
High school or less	10	52.6
College degree or less (Trade, AA, BA)	8	42.2
Master's or doctoral degree	1	5.3
Onboarded participants to myStrength	17	89.5
Attended at least one of the four-class technology training	15	78.9

*Note.* *N* = 19. Sociodemographic data is missing for one participant user, who chose not to complete a demographic survey. Pilot staff were on average 33 years old (*SD *= 12.4; range = 22–57).

#### Supporter Time

Staff cumulatively spent a total of 217 hr providing support to users during the 6-month pilot, with each staff member spending an average of 19.8 hr (*SD *= 16.51; range = 1–47). Support activities included logistics, user check-ins, digital literacy training, technology assistance, referrals, supervision, and translations. We categorized these activities into preparatory activities (i.e., activities that occurred prior to users’ use of myStrength) and service delivery and operation activities (i.e., activities that occurred during users’ use of myStrength). See Supplementary Appendix B for detailed definitions. In total, staff spent 64% of their time (140 hr) in preparatory activities, averaging about 22.4 hr (*SD *= 4.99) per week. Meanwhile, they spent 35% (77 hr) in activities related to service delivery and operations, averaging about 12.2 hr (*SD *= 2.73) per week. See Table 4 for a time breakdown.

**Table 4:** Frequency and Percentage of Time Spent Pilot Staff Spent Providing Human Support Across 6-Month Pilot by Activity.

Type of activity	Time spent	
	Frequency	%
Preparatory		
Logistics	46	21.0
Digital literacy training	94	43.0
Translations	1	0.0
Service delivery and operations		
User check-ins	29	13.0
Technological assistance	33	15.0
Referrals	11	5.0
Supervision	3	1.0

## Discussion

### Principal Findings

This evaluation demonstrated promise and challenges for using TESs in a county mental health service setting. Although users were not a one-to-one representation of Marin County demographics, the pilot met the goal to over select isolated older adults. This is crucial given that this population is typically not included in studies of TESs, less likely to receive mental health services, and more common in publicly funded service settings (Saeed & Masters, 2021; SAMHSA, 2020). We found reductions in loneliness, supporting the feasibility that myStrength paired with human support might impact loneliness in real-world settings, albeit with a small, but diverse, sample. Although we cannot disentangle what led to the reductions, myStrength itself, the human support, a combination, or whether loneliness changed on its own over time; this deployment is consistent with the notion of a TES. Users logged onto myStrength on average once per week to complete four activities, which is comparable with typical schedules for attending psychotherapy, but without the need to travel to an appointment. This engagement is relatively higher than what is typically found in real-world deployments of TESs (Baumel et al., 2019). As an example, some research suggests that most users of similar platforms only log in once and rarely complete any in-app activities (Fleming et al., 2018). However, we also note that use varied considerably among users, and better understanding the motivation and benefits obtained from such “super users” could be useful as explored in other areas of health technologies (e.g., Vandelanotte et al., 2022).

In assessing the support provided, we observed that staff adjusted their delivery of myStrength over time and across clients. It is noteworthy that the specific activities staff were expected to follow were not well codified at the beginning of the pilot. Staff received some general training on myStrength by the county and were given considerable autonomy, which might explain why a large majority of staff reported making some sort of modification in their delivery of myStrength. We also found that staff spent 217 hr providing support or about 1.2 hr per user per week. This suggests that although digital interventions may not result in less time spent to support delivery, they may facilitate task-shifting paradigms, whereby interventions are provided by non-specialists. This shift could enhance accessibility care (Naslund et al., 2019). In general, staff spent more time on preparatory than service delivery and operation activities. This finding is unsurprising given that although most participants were enthusiastic about receiving myStrength, they generally lacked the technical readiness to use it. Some of the challenges included lack of devices or Internet connection and low levels of digital literacy. As such, considerable effort was required to provide training, support, and resources before users could fully engage with the platform. This aligns with existing research, which indicates that individuals with lower socioeconomic status and limited digital skills often require more initial human support to effectively engage with new technologies and have a positive user experience (Hernandez-Ramos et al., 2021).

### Limitations

Findings should be interpreted based on the context of our evaluation. As a state-funded innovation project, our assessment strategy focused on pragmatism and minimizing participant burden. We used brief measures, collected information directly related to evaluation questions, and relied on hour logs provided by staff. Our data came from deployment in a single county and the sample size was based on the number of participants they deemed necessary for decision-making, not statistical power. As such, we did not conduct moderation analyses or subgroup comparisons given these analyses would be underpowered and not produce robust estimates. Future implementations of TESs, in both research and practice, could benefit from larger samples, longer evaluation periods, and longer and more diverse measures based on evaluation or research questions.

## Conclusions

Our findings can guide future implementations that seek to target underserved individuals, leverage TESs, and integrate models of human support. Recruiting the target population required a multipronged recruitment approach that leveraged existing community partnerships. Furthermore, given that previous research has shown that there is a diverse and wide range of training requirements for human supporters assisting with the implementation and service delivery of TESs (Bernstein et al., 2022), the fact that these supporters were nurses and *promotores* shows that individuals with various backgrounds and training can fill this roll. At the same time, the amount of time needed to provide this support suggests that integrating TESs into publicly funded service setting may need to be intensive and ongoing. To ensure that this support is effective and sustainable, future work in TES implementation should assess support needs of the target population from the start and build appropriate support components for both the technology and the context.

## Supplemental Material

sj-docx-1-irp-10.1177_26334895241288571 - Supplemental material for Evaluation of a pilot implementation of a digital cognitive behavioral therapy platform for isolated older adults in county mental health services Supplemental material, sj-docx-1-irp-10.1177_26334895241288571 for Evaluation of a pilot implementation of a digital cognitive behavioral therapy platform for isolated older adults in county mental health services by Rosa Hernandez-Ramos, Stephen M. Schueller, Judith Borghouts, Kristina Palomares, Elizabeth Eikey, Margaret Schneider, Nicole A. Stadnick, Kai Zheng, Dana B. Mukamel and Dara H. Sorkin in Implementation Research and Practice

sj-docx-2-irp-10.1177_26334895241288571 - Supplemental material for Evaluation of a pilot implementation of a digital cognitive behavioral therapy platform for isolated older adults in county mental health services Supplemental material, sj-docx-2-irp-10.1177_26334895241288571 for Evaluation of a pilot implementation of a digital cognitive behavioral therapy platform for isolated older adults in county mental health services by Rosa Hernandez-Ramos, Stephen M. Schueller, Judith Borghouts, Kristina Palomares, Elizabeth Eikey, Margaret Schneider, Nicole A. Stadnick, Kai Zheng, Dana B. Mukamel and Dara H. Sorkin in Implementation Research and Practice

sj-pdf-3-irp-10.1177_26334895241288571 - Supplemental material for Evaluation of a pilot implementation of a digital cognitive behavioral therapy platform for isolated older adults in county mental health services Supplemental material, sj-pdf-3-irp-10.1177_26334895241288571 for Evaluation of a pilot implementation of a digital cognitive behavioral therapy platform for isolated older adults in county mental health services by Rosa Hernandez-Ramos, Stephen M. Schueller, Judith Borghouts, Kristina Palomares, Elizabeth Eikey, Margaret Schneider, Nicole A. Stadnick, Kai Zheng, Dana B. Mukamel and Dara H. Sorkin in Implementation Research and Practice

## Notes

1. This pilot implementation was not pre-registered by the county. Furthermore, the data collected and analyzed during this evaluation are not publicly available due to contractual constraints.

2. Although the project was designed to recruit isolated older adults, no formal inclusion criterion was established for isolation. Instead, this was reflected in their project planning and selection of community partnerships.

3. It is unknown how many older adults were approached and declined participation as the county did not keep track of this in detail.

4. At the time of the pilot, the Spanish language version of myStrength had its content, but not its layout (i.e., headings) translated to Spanish.

5. Though not specifically developed for older adults, myStrength was selected based on previous exploratory focus group discussions with older adults in Marin County conducted in preparation for the grant.

6. Out of 30 platform users that enrolled in the pilot implementation, 28 completed Survey 1, 25 Survey 2, and 23 Survey 3. Additionally, three platform users did not fully answer all the items in the measures in Survey 1, Survey 2, and Survey 3, respectively.
