# Supplementary material for: Evaluation of a pilot implementation of a digital cognitive behavioral therapy platform for isolated older adults in county mental health services
Source: Implement Res Pract. 2024 Oct 15;5:26334895241288571. doi: 10.1177/26334895241288571 (PMC11489907; doi:10.1177/26334895241288571)
Supplement: sj-docx-1-irp-10.1177_26334895241288571 - Supplemental material for Evaluation of a pilot implementation of a digital cognitive behavioral therapy platform for isolated older adults in county mental health services [file sj-docx-1-irp-10.1177_26334895241288571.docx]

**Figure 4**

*Breakdown of Adaptations to Presentation, Discussion, and Service Delivery Of myStrength*
