# Supplementary material for: Evaluation of a pilot implementation of a digital cognitive behavioral therapy platform for isolated older adults in county mental health services
Source: Implement Res Pract. 2024 Oct 15;5:26334895241288571. doi: 10.1177/26334895241288571 (PMC11489907; doi:10.1177/26334895241288571)
Supplement: sj-docx-2-irp-10.1177_26334895241288571 - Supplemental material for Evaluation of a pilot implementation of a digital cognitive behavioral therapy platform for isolated older adults in county mental health services [file sj-docx-2-irp-10.1177_26334895241288571.docx]

**Appendix A**

*Definition of Activities*

| **Type of Activity** | **Area** | **Definition** |
| --- | --- | --- |
| Logistics | Preparatory | Onboarding participants, starting and ending Wi-Fi services, hand-offs, coordinating support from nurse interns and promotores, coordinating with 3rd-parties, etc. |
| Digital Literacy Training | Preparatory | Anything that is related to learning how to use a device. The ability to use information and communication technologies to find, evaluate, create, and communicate information, requiring both cognitive and technical skills. (e.g., how to us Zoom, signing up for myStrength, exploring app features, etc.) |
| Translations | Preparatory | Further translating myStrength content into a form of Spanish that the participants could understand. |
| User Feedback | Service Delivery and Operations | Gathering feedback from participants or checking in with participants. |
| Technical Assistance | Service Delivery and Operations | Anything that is related to having issues with technology. Aiding with technology such as televisions, computers, and software, typically aiming to help the user with a specific problem. (e.g., Wi-Fi issues, issues with logging into accounts, etc.) |
| Referrals | Service Delivery and Operations | Connecting participants to resources and mental health support. |
| Supervision | Service Delivery and Operations | Supporting and providing feedback to nurse interns and promotores. |
